# Endometriosis with an acute colon obstruction: a case report

**DOI:** 10.1186/s13256-015-0609-5

**Published:** 2015-06-26

**Authors:** David N Baden, Anthony van de Ven, Paul CM Verbeek

**Affiliations:** Department of General Surgery, Flevoziekenhuis, Hospitaalweg 1, 1315 RA Almere, the Netherlands

**Keywords:** Acute abdominal pain, Deep infiltrating endometriosis, Emergency surgery

## Abstract

**Introduction:**

The presentation of an acute bowel obstruction caused by endometriosis in an emergency department setting is rare, as it usually presents through years of complaints in the absence of a distinct acute onset. In this report, we present a case of a patient who was familiar with abdominal complaints and eventually required emergency surgery to treat an acute bowel obstruction caused by endometriosis. Endometrioses present infrequently in the acute phase, and only a few cases in which emergency surgery was required have been described in the literature.

**Case presentation:**

A 31-year-old Caucasian woman presented to the emergency room of our hospital with a distended abdomen, pain and nausea accompanied by a history of 14 years of chronic abdominal pain and constipation. An abdominal X-ray and subsequent computed tomographic scan showed a severely distended cecum of 9cm with stenosis in the sigmoid. Cecal blow-out was considered highly likely, and, during an emergency laparotomy, an obstructing process was found in the sigmoid. An oncologic resection of the sigmoid was performed with a primary anastomosis and loop ileostomy. A pathological examination revealed a tumor of 4cm in the sigmoid, which contained a tubelike structure with cytogenic stroma and the remains of focal bleeding. These are typical aspects of endometriosis.

**Conclusions:**

Infiltrating endometriosis is an invalidating disease that can be misdiagnosed for a wide range of other diseases. Emergency room physicians and surgeons should be aware that it can present as an acute obstruction and should be considered in diagnosing women of childbearing age. After initial colonoscopy, emergency surgery is the best therapeutic approach if there is a complete obstruction.

## Introduction

In this case report, we describe a patient who had a long history of abdominal complaints. She came to our emergency department (ED) with a rare case of acute bowel obstruction caused by endometriosis. The diagnosis of endometriosis is usually made after years of complaints with the absence of a distinct acute onset [1,2].

## Case presentation

A 31-year-old Caucasian woman with increasing abdominal distension, pain and nausea was referred to our department by gastroenterologists at the ED. She had had abdominal pain and chronic constipation symptoms since the age of 17 years. These complaints had been extensively analyzed several years earlier by the gastroenterology department, when no abnormalities were identified in blood tests or a colonoscopy. One month prior to her presentation at the ED, she had been admitted to the surgical department with complaints of decreased intake, nausea and a 10-day absence of defecation. Blood tests did not show abnormalities. An abdominal X-ray showed signs of constipation. After conservative treatment with enemas and oral debulking agents, her complaints decreased and she was dismissed from hospital.

During the intake at the ED, she was found to have no history of weight loss, and, with the use of bisacodyl and close monitoring of her diet, she had managed to regulate her bowel movements, with the exception of her recent admission. For the preceding 1 year, the patient had desired to become pregnant. Therefore, she had stopped using oral contraceptives, after which she had a regular cycle without intermediate bleeding or abnormal vaginal discharge.

**Figure 1:**
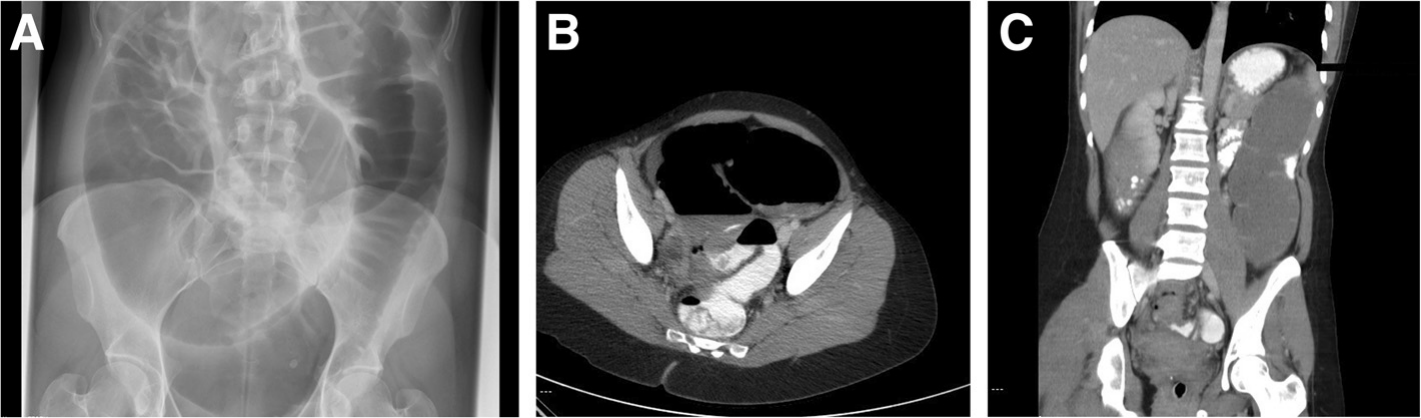
Pre-operative imaging studies. X-ray **(A)** and computed tomographic scans **(B, C)** of the patient’s abdomen show a significantly inflated cecum and an obstruction in the sigmoid colon with a proximal distension of the colon.

In her physical examination, we encountered a painful woman with a normal body temperature. She had abdominal distension, hypertympanic percussion, active bowel movements and tenderness of the abdomen without signs of abdominal guarding. Additional blood tests showed a normal hemoglobin level of 7.8 (normal range, 7 to 10mmol/l), without any signs of an infection with 6.8×10^4^ leukocytes (normal range, 5 to 10×10^4^) and a C-reactive protein level of 8.0 (normal level, <10mg/l). An abdominal X-ray (Figure 1A) and subsequent abdominal computed tomography (CT) (Figure 1B,C) showed a significantly inflated cecum of 9cm and an obstruction in the sigmoid colon with a proximal distension of the colon. Because of the expansion of the cecum, a decision was made to perform emergency colonoscopy for desufflation; however, the obstruction could not be passed with a scope.

Given the threat of cecal blow-out, it was decided to perform a laparotomy. During the surgical procedure, a solitary stenosis was found in the sigmoid colon that appeared as a malignancy. Therefore, an oncological sigmoid resection with primary anastomosis was performed. Because of the distension of the cecum, a temporary loop ileostomy was constructed. Upon further inspection of the abdomen, no abnormalities were found in the liver, peritoneum or pelvis. The patient recovered quickly after the procedure and could be discharged after 1 week, with the intention to perform a loop ileostomy reversal after 3 months.

**Figure 2:**
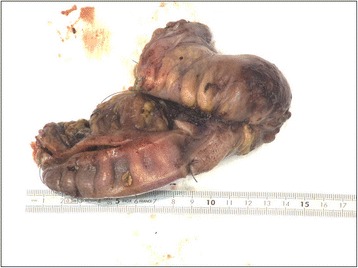
Pathological specimen obtained during sigmoid resection.

**Figure 3:**
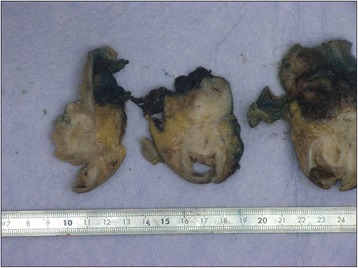
Extensive fibrotic foci can be seen with induration in the intestinal wall without mucosal abnormalities.

**Figure 4:**
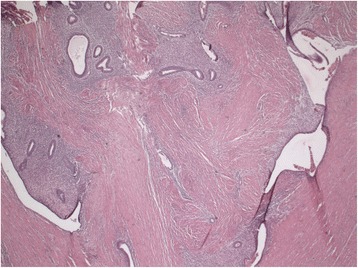
Microscopically, the tumor had epithelial tubular structures surrounded by arguably cytogenetic stroma and focal hemorrhage remnants suggestive of endometriosis.

A macroscopic pathological examination of the resected sigmoid showed extensive fibrotic foci with induration in the intestinal wall without mucosal abnormalities (Figures 2 and 3). In the sigmoid wall, a tumor 4.4cm in size was found; however, this did not appear to have an obvious relationship to the mucosa. Microscopically, the tumor showed epithelial tubular structures surrounded by what was interpreted as cytogenic stroma and focal hemorrhage remnants, suggestive of endometriosis (Figure 4). Foci of tubes with ciliated columnar epithelium were present in some lymph nodes.

## Discussion

*Endometriosis* is defined as the implantation of endometrium outside the uterus [3,4]. In 1927, Sampson theorized that endometriosis results from retrograde deposition of endometrial remains during menstruation [5]. These endometrial cells remain sensitive to gonadotropic hormones and react to hormonal changes during the menstrual cycle. Sampson’s theory does not fully explain why endometriosis occurs only in a select group of women, and to date the exact etiology remains unknown. Recent research has shown that differences in genetic and immunological factors exist between affected and unaffected women [1,6,7].

Endometriosis affects 10% to 12% of fertile women, with the number of cases diagnosed peaking between the ages of 29 to 39 years. First onset can be as early as adolescence, making it prone to being missed as a diagnosis [1-3,8,9]. Endometriosis can occur in the entire abdominal cavity and is often classified into three groups: peritoneal endometriosis, ovarian endometriosis and deep infiltrating endometriosis [10]. Deep infiltrating endometriosis is not clearly defined, but usually it is described as an ingrowth of over 5mm into the peritoneum and underlying structures [1,4,7,10]. In 5% to 12% of the women affected by endometrioses, there is infiltrating growth in the rectosigmoid [2,3,10,11]. Additional common locations for ingrowths are the uterosacral ligaments, vagina, bladder and ureters [10].

Symptoms of deep infiltrating endometriosis of the rectosigmoid include dysmenorrhea, constipation, diarrhea, pain during defecation, dyspareunia and periodic rectal bleeding during menstruation [2,3,10,12,13]. Late presenting symptoms can be chronic because of extensive fibrosis that occurs as a result of the endometriosis depositions. Physical examination often provides insufficient clues to lead to suspicion of deep infiltrating endometriosis; hence, imaging is an important aspect of making the diagnosis [4,7]. To diagnose deep infiltrating endometriosis, transvaginal ultrasound is a highly reliable imaging technique to complement the gold standard of laparoscopy [1,3,4]. Transvaginal ultrasound has a reported sensitivity of 91% and specificity of 98% [14]. For large depositions in the cavum Douglasi, and as pre-operative imaging, a magnetic resonance imaging scan with rectal contrast has additional value and is superior to CT [3,15].

Deep infiltrating endometriosis often presents at a late stage with extensive complaints due to extensive fibrosis caused by chronic inflammation of the endometriosis. Anti-inflammatory and hormonal therapies have little to no effect on the symptoms and therefore provide no added value in this stage of the disease [10]. Surgical intervention has been argued to be the treatment of choice at this stage. In recent years, a number of new techniques have been developed; however, the gold standard remains segmental resection laparoscopy or, if this is complicated because of adhesions, laparotomy [2,11,16]. In this otherwise healthy patient population, fewer complications are seen than with oncological colorectal surgery [17]. The most frequent complications are rectovaginal fistula (2% to 8%) and anastomotic leakage (0 to 3%). Similarly to oncological colorectal surgery, there is a strong link between the risk of anastomotic leakage and the position of the anastomoses [2,10,11,13,17]. There is less need for the construction of loop ileostomy in this group of healthy young women. This decision making should additionally take into consideration the severe psychological effects that accompany a stoma at this age [17]. A partial resection or curettage of the lesion is an alternative to a bowel resection with a lesion smaller than 2cm [2,4,10]. The risk of recurrence after an intervention for infiltrating endometriosis varies widely and is difficult to assess because of differing research methods and follow-up durations. Most review authors state a recurrence risk of 5% to 25% [2,10,11]. Remarkably, this is not influenced by the radicality of the resection [12].

## Conclusions

This case illustrates that infiltrating endometriosis is a disabling disease. Emergency room physicians and surgeons should be aware that it can present as an acute obstruction and should be considered in diagnosing women of childbearing age. In our patient, a CT scan, rather than an abdominal X-ray, might have revealed the pending obstruction in an earlier phase. In exceptional cases, colon obstruction by infiltrating endometriosis can require an emergency laparotomy with resection of the affected bowel segment. In less urgent cases, more planning is recommended, along with discussion of the case, including medical images, among a multidisciplinary endometriosis team.

## Consent

Written informed consent was obtained from the patient for publication of this case report and any accompanying images. A copy of the written consent is available for review by the Editor-in-Chief of this journal.

## Abbreviations

CT: Computed tomography
ED: Emergency department

## References

[1] Luisi S, Lazzeri L, Ciani V, Petraglia F. Endometriosis in Italy: from cost estimates to new medical treatment. Gynecol Endocrinol. 2009;25:734–40. 10.3109/09513590903159664 19908951

[2] Koh CE, Juszczyk K, Cooper MJ, Solomon MJ. Management of deeply infiltrating endometriosis involving the rectum. Dis Colon Rectum. 2012;55:925–31. 10.1097/DCR.0b013e31825f3092 22874598

[3] Nassif J, Trompoukis P, Barata S, Furtado A, Gabriel B, Wattiez A. Management of deep endometriosis. Reprod Biomed Online. 2011;23:25–33. 10.1016/j.rbmo.2010.08.012 21474383

[4] Daraï E, Bazot M, Rouzier R, Houry S, Dubernard G. Outcome of laparoscopic colorectal resection for endometriosis. Curr Opin Obstet Gynecol. 2007;19:308–13. 10.1097/GCO.0b013e328216f6bc 17625410

[5] Sampson JA. Metastatic or embolic endometriosis, due to the menstrual dissemination of endometrial tissue into the venous circulation. Am J Pathol. 1927;3:93–110.43. PMC1931779 19969738

[6] Viganò P, Parazzini F, Somigliana E, Vercellini P. Endometriosis: epidemiology and aetiological factors. Best Pract Res Clin Obstet Gynaecol. 2004;18:177–200. 10.1016/j.bpobgyn.2004.01.007 15157637

[7] Koninckx PR, Ussia A, Adamyan L, Wattiez A, Donnez J. Deep endometriosis: definition, diagnosis, and treatment. Fertil Steril. 2012;98:564–71. 10.1016/j.fertnstert.2012.07.1061 22938769

[8] Bascombe NA, Naraynsingh V, Dan D, Harnanan D. Isolated endometriosis causing sigmoid colon obstruction: a case report. Int J Surg Case Rep. 2013;4:1073–5. 10.1016/j.ijscr.2013.09.015 PMC3860036 24240070

[9] Roman H, Ness J, Suciu N, Bridoux V, Gourcerol G, Leroi AM, et al. Are digestive symptoms in women presenting with pelvic endometriosis specific to lesion localizations? A preliminary prospective study. Hum Reprod. 2012;27:3440–9. 10.1093/humrep/des322 22962316

[10] Meuleman C, Tomassetti C, D’Hoore A, Van Cleynenbreugel B, Penninckx F, Vergote I, et al. Surgical treatment of deeply infiltrating endometriosis with colorectal involvement. Hum Reprod Update. 2011;17:311–26. 10.1093/humupd/dmq057 21233128

[11] De Cicco C, Corona R, Schonman R, Mailova K, Ussia A, Koninckx P. Bowel resection for deep endometriosis: a systematic review. BJOG. 2011;118:285–91. 10.1111/j.1471-0528.2010.02744.x 21040395

[12] Mabrouk M, Spagnolo E, Raimondo D, D’Errico A, Caprara G, Malvi D, et al. Segmental bowel resection for colorectal endometriosis: is there a correlation between histological pattern and clinical outcomes? Hum Reprod. 2012;27:1314–9. 10.1093/humrep/des048 22416007

[13] Jelenc F, Ribič-Pucelj M, Juvan R, Kobal B, Sinkovec J, Salamun V. Laparoscopic rectal resection of deep infiltrating endometriosis. J Laparoendosc Adv Surg Tech A. 2012;22:66–9. 10.1089/lap.2011.0307 22166117

[14] Hudelist G, English J, Thomas AE, Tinelli A, Singer CF, Keckstein J. Diagnostic accuracy of transvaginal ultrasound for non-invasive diagnosis of bowel endometriosis: systematic review and meta-analysis. Ultrasound Obstet Gynecol. 2011;37:257–63. 10.1002/uog.8858 20954166

[15] Scardapane A, Lorusso F, Bettocchi S, Moschetta M, Fiume M, Vimercati A, et al. Deep pelvic endometriosis: accuracy of pelvic MRI completed by MR colonography. Radiol Med. 2013;118:323–38. 10.1007/s11547-012-0850-6 22744354

[16] de Jong MJ, Mijatovic V, van Waesberghe JH, Cuesta MA, Hompes PG. Surgical outcome and long-term follow-up after segmental colorectal resection in women with a complete obstruction of the rectosigmoid due to endometriosis. Dig Surg. 2009;26:50–5. 10.1159/000194197 19155628

[17] Ruffo G, Sartori A, Crippa S, Partelli S, Barugola G, Manzoni A, et al. Laparoscopic rectal resection for severe endometriosis of the mid and low rectum: technique and operative results. Surg Endosc. 2012;26:1035–40. 10.1007/s00464-011-1991-8 22038165

